# Identification and isolation of embryonic stem cells in reproductive endocrinology: theoretical protocols for conservation of human embryos derived from *in vitro *fertilization

**DOI:** 10.1186/1742-4682-2-25

**Published:** 2005-07-18

**Authors:** Eric Scott Sills, Takumi Takeuchi, Noriko Tanaka, Queenie V Neri, Gianpiero D Palermo

**Affiliations:** 1 Georgia Reproductive Specialists LLC, Division of Reproductive Endocrinology and Infertility, Department of Obstetrics and Gynecology, Atlanta Medical Center; Atlanta, Georgia 30342 USA; 2 Cornell Center for Reproductive Medicine and Infertility, Weill Medical College of Cornell University, New York, New York 10021 USA; 3 HT-336, 505 East 70^th ^Street, New York, New York 10021 USA

## Abstract

**Background:**

Embryonic stem cells (ESC) are pluripotent cells obtained from the inner cell mass (ICM) of blastocysts derived from *in vitro *culture associated with reproductive endocrinology therapy. Human ESCs are regarded as highly significant since they retain the capacity to differentiate into any of approximately 200 unique cell types. Human ESC research is controversial because to acquire such cells, the ICM of human blastocysts must be manipulated in a way that renders embryos nonviable and unsuitable for transfer *in utero*. Techniques to yield competent ESCs with conservation of source blastocysts would satisfy many objections against ESC research, but at present such approaches remain largely untested.

**Results and discussion:**

We contrast experimental culture of single blastomeres obtained by 1) non-destructive biopsy of embryos destined for transfer, and 2) isolation of karyotypically normal blastomeres from disaggregated ("dead") embryos considered unsuitable for transfer, and evaluate these approaches with regard to production of ESCs. Pluripotency was confirmed by morphological criteria and by quantification of divergent homeodomain proteins specific to undifferentiated cell development. Following ESC isolation and identification, assessment was conducted according to a novel ESC grading system, also proposed here.

**Conclusion:**

The role of reproductive endocrinology in ESC research remains paramount. In this report, we hypothesize new and expand on existing strategies having the potential to enhance human ESC isolation, identification and *in vitro *maintenance.

## Background

While the definitive characterization of murine embryonic stem cells was first reported in 1981, embryonic stem cells (ESC) were not isolated and fully described in humans until much later [1]. Without question, the scarce supply of human ESCs combined with the technical challenges associated with interspecies translation of stem cell derivation contributed to the long interval between these reports. To obtain human ESCs, embryos produced during *in vitro *fertilization (IVF) are maintained in extended culture to the blastocyst stage (4–5d post fertilization) when the polarized inner cell mass (ICM) develops. The outer trophoectoderm is removed via immunosurgery, thus exposing the ICM for disaggregation and plating on a feeder cell layer for further culture. Importantly, this disruptive process renders the embryo non-viable and unsuitable for *in utero *transfer [2]. Homogenous human ESC colonies may be derived from subsequent isolation and re-plating of the ICM cells, which are then screened for stemness by a variety of recognized markers.

Once in stable culture, ESCs are capable of either symmetric (clonogenic) or asymmetric fission. Symmetric ESC division yields a self-renewing supply of pluripotent ESCs, while asymmetric division produces one cell identical to the parent ESC plus one differentiated cell. The mechanism(s) responsible for modulating these specific ESC fission patterns remain poorly understood. In any case, since it is not yet possible to de-differentiate committed somatic cells to reacquire pluripotency, embryos associated with IVF have thus far been the only source for human ESCs. The fact that live human embryos must be destroyed to produce human ESCs presents a substantial ethical obstacle for the advancement of human ESC research. With the vast therapeutic promise of human ESC seen against the destruction of human embryos required to realize such aspirations, compelling arguments have been articulated both in support of and in opposition to human ESC research [3,4].

It must be admitted that thus far human ESCs have provided no reproducible, safe, unique and previously unattainable treatment for any human disease. Nevertheless, interest in exploration of the full therapeutic possibility of human ESCs continues to grow and is no longer confined to medical scientists and reproductive endocrinologists – indeed, it now includes public opinion leaders and medical consumers as well [5]. Yet the concerns of human ESC research opponents are not without ethical justification; these objections could be substantially assuaged if safe and effective laboratory protocols could be developed that offered human ESCs whilst preserving (or at least not destroying) the blastocysts from which they originated. In this paper we present results from pilot studies based on some theoretical approaches, in a manner to facilitate human ESC research and to promote respect for human embryos obtained from clinical reproductive endocrinology practice.

### Human embryonic stem cells: theoretical approaches

#### Blastomere biopsy and culture

Prior to the blastocyst stage, a human embryo at 2–3d post fertilization consists of just 4–8 cells, all of which are totipotent. In contrast to the pluripotent cells obtained from the blastocyst ICM, one or two of these blastomeres may be biopsied without compromising the integrity of the sampled embryo [6]. Blastomeres obtained for PGD are generally fixed and processed with fluorochromes to detect aneuploidy by partial karyotype analysis, although the process has more recently advanced to testing for single gene disorders via polymerase chain reaction [7] and single cell whole genome amplification by multiple displacement amplification [8]. Such processing irrevocably alters the blastomere destined for PGD – the viability of this cell is sacrificed in return for the vital genomic information provided by PGD. However, assuming two distinct blastomeres were extracted at a well-timed embryo biopsy for PGD, and since in the absence of mosaicism each blastomere retains the potential to develop into a complete organism [9], the possibility exists that at least one sampled blastomere obtained for PGD could be maintained in culture specifically for ESC production. As with traditional PGD protocols, genetic data needed from PGD could still be obtained and inform embryo transfer decisions, while the second blastomere could provide a potential source of human ESCs with no measurable adverse affect on the developmental integrity of the biopsied embryo.

**Figure 1:**
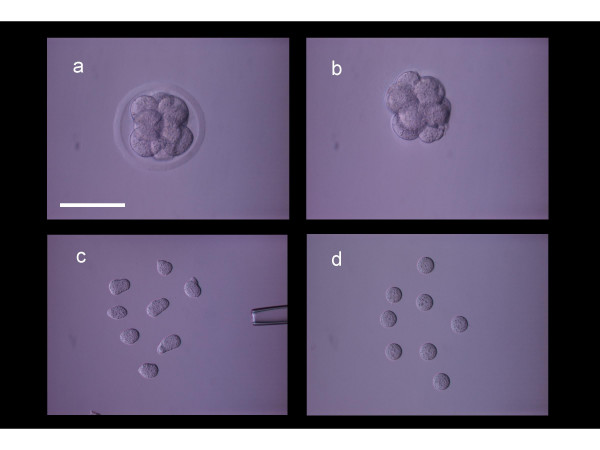
Blastomere isolation sequence. An intact 8-cell mouse embryo (a) was subjected to pronase digestion to remove the zona pellucida (b). Single blastomeres were disaggregated by microdissection (c) and after stabilization in culture were monitored for further treatment (d). Scale bar = 100 microns.

**Figure 2:**
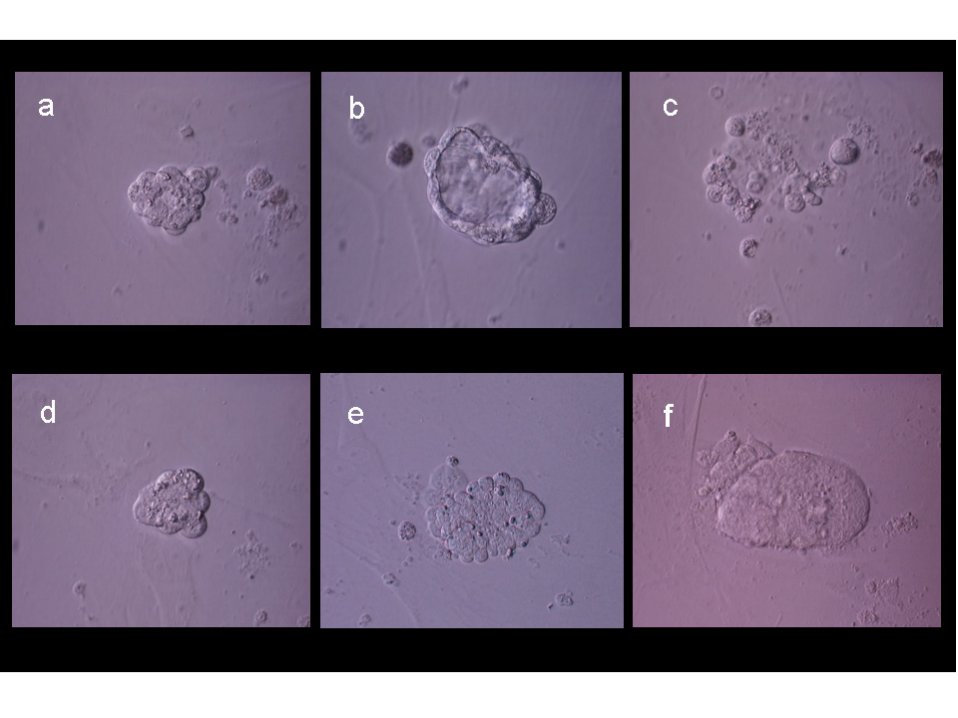
Evolution of experimental blastomere growth observed on feeder cell layer on culture days 2, 3, and 4. Top row shows a single blastomere undergoing cleavage (a) and forming a "unilaminar vesicle" on day 3 (b). Cellular arrest and degeneration were evident by day 4 (c). Bottom row shows another cleaving blastomere (d), which formed a cellular aggregate on day 3 (e) and later developed an inner cell mass-like structure (f).

Utilizing a murine embryo model, we evaluated this concept where two blastomeres were isolated from a single 8-cell embryo via standard microsurgical biopsy techniques [10]. Zona-free murine blastomeres were then washed, individually plated and cultured as previously described [11]; embryos from which the biopsies were taken were maintained in standard culture (control group). All cells were monitored × 12 h to assess cleavage, differentiation, and attachment to the feeder cell monolayer, as applicable (Figure 1 and 2). With proper culture conditions we observed advancement to morphologically normal blastocyst stage in both groups. Next, cells resembling an ICM that originated from the intact/source embryo group and the single blastomere culture group were disaggregated from their respective blastocysts and re-plated on to fresh feeder cells for confirmation and further analysis; no cells were frozen. This work carries forward a theoretical approach suggested more than a decade ago [12], and demonstrates that a blastomere biopsy and culture approach can supply a single totipotent cell for subsequent ESC culture without harming the source embryo.

#### Blastomere donation from non-viable ("dead") embryos

Human embryo assessment plays a central role in IVF to identify embryos with the best prognosis for transfer, but what is less clear is the fate of embryos judged not suitable for transfer or cryopreservation due to arrested growth or gross developmental abnormality. Despite the absence of formal guidelines governing human embryology practice, many IVF centers carefully monitor embryos over several days before making the determination that they should be neither transferred nor cryopreserved based on non-viability. Indeed, even with cryopreservation as late as post-fertilization day 7, human livebirths have been achieved [13]. However, as previous investigators have noted [2,14], a consensus definition of embryo non-viability or death remains elusive and it is reasonable to expect that the concept of embryo death will formalize gradually in a process similar to that which led to the 1981 Uniform Determination of Death Act [15]. In the meantime, most major IVF clinics already obtain written informed consent from patients to discard any human embryos deemed non-viable or dead.

Interestingly, IVF laboratories have confronted this challenge and produced an informal if not exactly uniform process to declare a human embryo "dead". Since the life of any developing organism is more than the sum of its cellular parts, it has been suggested that the defining vital characteristics of a 4- or 8-cell human embryo must include continued and integrated cellular division, growth, and differentiation [16]. And by extension, embryos that have irreversibly lost this basic capacity (even if individual constituent cells may remain alive) should be properly regarded as organismically dead. Therefore our investigations were based on assessment of fresh (non-cryopreserved) 4–8 cell embryos demonstrating developmental arrest observed over an 8-day *in vitro *culture interval. Among such non-viable embryos destined for discard, a high rate of chromosomal error has been found in some, but not all, blastomeres [17]. It is the salvage of any normal blastomeres within a "dead" embryo that holds particular promise for human ESC research. Specifically, if embryos classified as non-viable and unsuitable for transfer or cryopreservation were disaggregated (rather than discarded) and plated as single totipotent blastomeres as described above, then the possibility exists that at least some karyotypically normal cell colonies could develop and serve as a reliable human ESC source. While the attempt to produce blastocysts from isolated blastomeres *in vitro *is not new [18], we feel this approach has received limited attention and merits further exploration to advance human ESC research, particularly since this source of ESCs would not derive from human embryos otherwise destined for transfer or cryopreservation.

We investigated the efficacy of a novel methodology with murine embryos that failed to meet viability standards, and were therefore unsuitable for transfer or cryopreservation. Embryos used in this pilot study displayed arrested growth and were classified as nonviable no later than the 8-cell stage. Embryos were disaggregated into single blastomeres by brief exposure to trypsin under micromanipulation control. Next, blastomeres were individually plated on a feeder cell layer and cultured in an experimental medium supplemented with β-mercaptoethanol, amino acids, nucleosides, antibiotics, L-glutamine with 2000 IU/ml mouse recombinant leukemia inhibiting factor in 6% CO_2 _at 37°C. Fully-expanded or hatching mouse blastocysts were plated as controls. The salvaged blastomeres and normal blastocysts were monitored daily to evaluate differentiation, cleavage and attachment to the feeder cell layer. Although some blastomeres obtained from the dead embryos failed to progress, a few ICM-like clusters developed from single blastomeres. These were isolated (as were ICMs derived from the intact blastocysts) and disaggregated into single cells by trypsinization and replated on to fresh feeder cell layers. These ESC lines were assessed for pluripotency by morphological criteria as well as alkaline phosphatase activity, Oct-4, and TROMA-1 [19], which validated stemness in this experiment.

### Impact of mosaicism on ESC derivation

Soon after the first clinical experience with preimplantation genetic diagnosis was reported [20], it was suggested that blastomere mosaicism might contribute to the clinical error rate observed in PGD [21]. The precept that not all blastomeres are necessarily equivalent has subsequently emerged as a recognized tenet in human embryology; it figures prominently in the informed consent for patients contemplating PGD [22]. Currently, a technique to determine the extent of embryo mosaicism without disassembling the embryo (and thus rendering it nonviable) does not exist. Accordingly, mosaicism presents potentially serious weaknesses for the two proposed ESC techniques described here, since the effectiveness of each approach is affected by the extent of blastomere mosaicism, which cannot be known *a priori*.

Nevertheless, for the two theoretical ESC protocols we present, the impact of embryo mosaicism is not the same and each instance deserves separate consideration. For example, if the PGD+blastomere biopsy and culture method were applied to embryos with extensive blastomere heterogeneity, this approach would be unlikely to produce chromosomally normal cells for subsequent *in vitro *ESC culture. If, however, embryos with very limited or no mosaicism are used for the proposed PGD+blastomere culture process, human ESC production could proceed with much greater likelihood given the uniformity of all sampled cells. Given the unknown extent of embryo mosaicism, limitations of single blastomere biopsy have been recognized [22] and some researchers have recommended confirmatory PGD by sampling a second blastomere [23]. In contrast, among blastomeres obtained from the disaggregation of nonviable embryos, it would be reasonable to expect a higher frequency of mosaicism. In such a setting, even limited mosaicism would yield the desirable result based on the presence of at least one genetically normal constituent blastomere within an organismically dead embryo.

### Objective assessment of ESC colonies

Although considerable resources are required to harvest and propagate ESCs, effective methods to verify stemness and monitor quality in such cells are also needed to bring the full range of therapeutic possibility into focus. In an effort to develop an assessment system for ESCs, our center cultured murine blastocysts on mouse fibroblasts in experimental media supplemented with 2000 IU/ml mouse recombinant leukemia inhibitory factor. At 4–5d, the ICMs were mechanically isolated and disaggregated by trypsin. Cell passages were repeated × 2–3d as needed, according to colony confluency.

**Figure 3:**
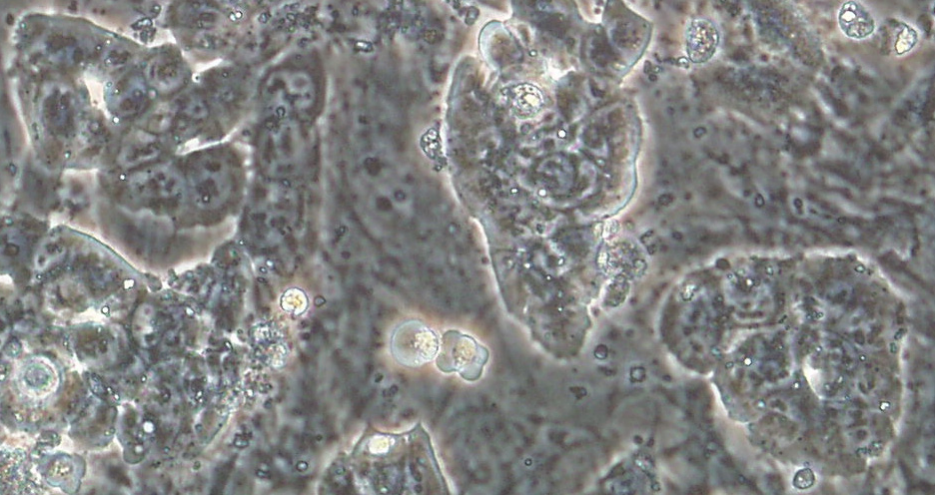
Experimental embryonic stem cell colonies derived from a single blastomere.

Our ESC colonies were then graded on the basis of three factors: 1) colony number, 2) colony density, and 3) colony quality. We determined colony character by morphological assessment using an inverted microscope with phase-contrast optics. Typically, ESCs are large and demonstrate a high nuclear:cytoplasm ratio (Figure 3). Each colony was classified according to the proportion of stem cells present within the colony, where >70% (good), 40–70% (average), or <40% (poor) were the three divisions. For all ESC colonies, alkaline phosphatase activity and Oct-4 were used as markers of totipotency. TROMA-1 antibody (monoclonal) directed against cytokeratin-like filaments of trophectoderm and endodermal cells served as a negative marker. As an additional control these markers were tested on expanded blastocysts. Specimens were fixed with 4% paraformaldehyde and permeabilized with 0.2% Triton X-100. Alkaline phosphatase activity in fixed cells was detected via azo-dye with Texas-red filter under fluorescence microscopy. ESCs were exposed to Oct-4 polyclonal antibody (1:100 dilution) and monoclonal TROMA-1 antibody (1:6 dilution), followed by rinse with PBS/BSA to remove unbound antibody. From these experiments, we obtained 16 murine ESC lines from 164 source blastocysts. Assessments via alkaline phosphatase and Oct-4 to verify pluripotency of the ESC lines were in agreement (χ^2 ^= 0.105), while TROMA-1 identified endoderm and trophoblast. Pluripotency was successfully confirmed in at least some cells from each colony studied, and we were able to establish concordance between morphological criteria and marker activity. Further studies will be helpful to show if additional parameters can refine this ESC scoring system.

### Stem cell research: social and political factors

Public sharing of information about the basic science of ESCs has proven to be important, since those who are aware of the stem cell debate tend to be more supportive of research in this area compared to those less familiar with the topic [24]. A national public opinion study (*n *= 1,512) conducted in 2004 found that a narrow majority (52%) of Americans regard the advancement of ESC research as more important than embryo destruction; when this question was posed just two years earlier, support for ESC research was 43% [24].

Discussion on human ESC research occurs against a stormy sociopolitical background – a circumstance not unlike earlier breakthroughs in sperm banking, oral contraception, *in vitro *fertilization, and intracytoplasmic sperm injection. For ESC, the debate was vocal and escalated quickly to the highest levels of government. In response to the 2005 Presidential State of the Union address, some leaders in the U.S. Congress have proposed standards to authorize broader use of human embryos in research. Specifically, legislation is planned to permit research involving human embryos but only if such embryos have been "ethically derived" – *i.e*. embryos developed for the purpose of IVF that would otherwise be discarded [25]. This legislative initiative is endorsed by the Coalition for the Advancement of Medical Research, Association of American Universities, Juvenile Diabetes Research Foundation, and Parkinson's Action Network, as well as the Cancer Research and Prevention Foundation.

This type of federal research funding strategy involving existing embryonic stem cell lines is consistent with the President's belief in the fundamental value and sanctity of human life. The President's decision reflects a commitment to preserve the value and sanctity of human life balanced with a desire to promote vital medical research. The Executive Order permits federal funding of research involving the more than 60 existing stem cell lines, but will not sanction or encourage destruction of additional human embryos. Indeed, the embryos from which the existing ESCs originated have already been destroyed. Federal funding of medical research on these existing stem cell lines will promote the sanctity of life "without undermining it" and will allow scientists to explore the potential of this research to benefit the lives of millions of people who suffer from life destroying diseases.

Only certain cell lines will be considered eligible for federally-sponsored human ESC research. These cell lines must be derived (1) with the informed consent of the donors, (2) from non-transferred embryos created solely for reproductive purposes, and (3) without any financial inducements to the donors. In order to ensure that federal funds are used to support only ESC research that is scientifically sound, legal, and ethical, the NIH will examine the derivation of all existing stem cell lines and create a registry of those lines that satisfy these criteria. Thus far, 23 ESC lines have proven viable and have met the NIH criteria, although up to 60 existing ESC lines from genetically diverse populations are eligible for federally-funded research [26].

## Conclusion

While other investigators have noted that it is not currently possible to transform a single blastomere into stem cells without recourse to formation and intentional destruction of whole blastocysts [16], in our studies *de novo *embryos were not generated. Indeed, only ICM-like cell clusters were obtained for further analysis. Refinement and increased efficiency of these two ESC protocols brings the potential to offer a reliable supply of embryonic stem cells without production of whole embryos or compromising extant source blastocysts otherwise selected for embryo transfer or cryopreservation.

Our work confirms the importance of identifying factors that facilitate growth and inhibit differentiation of ESCs. Liberation from a feeder cell requirement may be essential for certain types of experiments, as well as for production of cells for transplantation [27]. Despite extensive experience with mouse embryonic fibroblast feeder cell layers for "culture support", exactly what these feeder cells provide for ESCs has not yet been characterized at the molecular level. Microarray technology applied to early embryo biology promises to answer many of these questions. However, as continued effort is applied toward elucidating these mediators, an adequate supply of source ESCs will prove pivotal to the entire process. From this perspective, our work in a murine model offers some novel approaches to allow progress in ESC research that would not damage or destroy extant human embryos. It is hoped that further study of embryo biopsy+culture as well as blastomere donation from non-viable embryos will permit ethical ESC research to reach its full potential.

## Competing interests

The author(s) declare that they have no competing interests.

## Authors' contributions

ESS, TT, NT, QVN and GDP contributed equally to this work. GDP conceived of the project, coordinated the research, and edited the manuscript.
